# Posterior Tibialis Tendon Dislocation: Case Report and Review of Literature

**DOI:** 10.7759/cureus.19301

**Published:** 2021-11-06

**Authors:** Antigoni Gkoudina, Georgios Graikos, Maria Chatziargiriou, Panagiotis Saloupis

**Affiliations:** 1 Orthopaedic Department, General Hospital of Pella, Edessa, GRC; 2 Orthopedics and Traumatology, George Papanikolaou General Hospital of Thessaloniki, Thessaloniki, GRC; 3 Orthopedics and Traumatology, Ippokrateio Hospital of Thessaloniki, Thessaloniki, GRC

**Keywords:** tendon, posterior tibialis, tendon surgery, ankle sprain, dislocation

## Abstract

Posterior tibialis tendon (PTT) dislocation is an extremely rare yet significant finding in cases with recalcitrant pain over the medial malleolus, usually as a consequence of trauma. The diagnosis is frequently delayed, as the patient’s initial clinical presentation generally resembles benign musculoskeletal pathology of the ankle joint. Herein, we report the case of a female patient diagnosed with PTT dislocation after four weeks of conservative management for an ankle sprain. Surgical intervention, including retromalleolar groove-deepening and repair of flexor retinaculum with intraosseous suture anchors, proved to be successful after a mean follow-up of 12 months. A thorough literature review was conducted regarding the aforementioned injury, concluding that PTT dislocation-in spite of its rarity-should be included in the differential diagnosis of patients with importunate pain on medial malleolus after an ankle injury.

## Introduction

Dislocation of a posterior tibialis tendon (PTT) represents an exceptionally rare entity in the annals of orthopedics, with only a few cases reported hitherto in the literature [1]. The vast majority of the cases reported have been managed surgically; however, a reference operating method is yet to be established with conservative treatment not being the option of choice due to its ineffectiveness and poor outcomes [2]. Late recognition of PTT dislocation is frequent due to the rarity of the injury, and the preliminary diagnosis of an acute ankle sprain is at first made by the clinician. Accurate diagnosis is established later because of an ongoing pain over the medial malleolus that has to be further scrutinized [3]. We present a case of a 60-year-old female's PTT dislocation caused by an indirect injury mechanism. 

## Case presentation

Α 60-year-old female housewife presented to the emergency department complaining of acute pain at the medial malleolus of the right ankle. The mechanism of injury was vague as the patient described that the pain suddenly started as she bent over to lift a heavy box with her knees extended. She also mentioned a “snap” sensation at the time of injury, followed by an inability to bear weight.

During the physical examination, moderate edema, ecchymosis, and tenderness were noted over the medial malleolus with a full range of ankle motion. Plain radiographs (anteroposterior, lateral, and mortise view) were normal. The patient was diagnosed with an acute sprain of the deltoid ligament and was managed conservatively with a non-weight bearing air cast, early functional rehabilitation, and nonsteroidal anti-inflammatory drugs.

**Figure 1:**
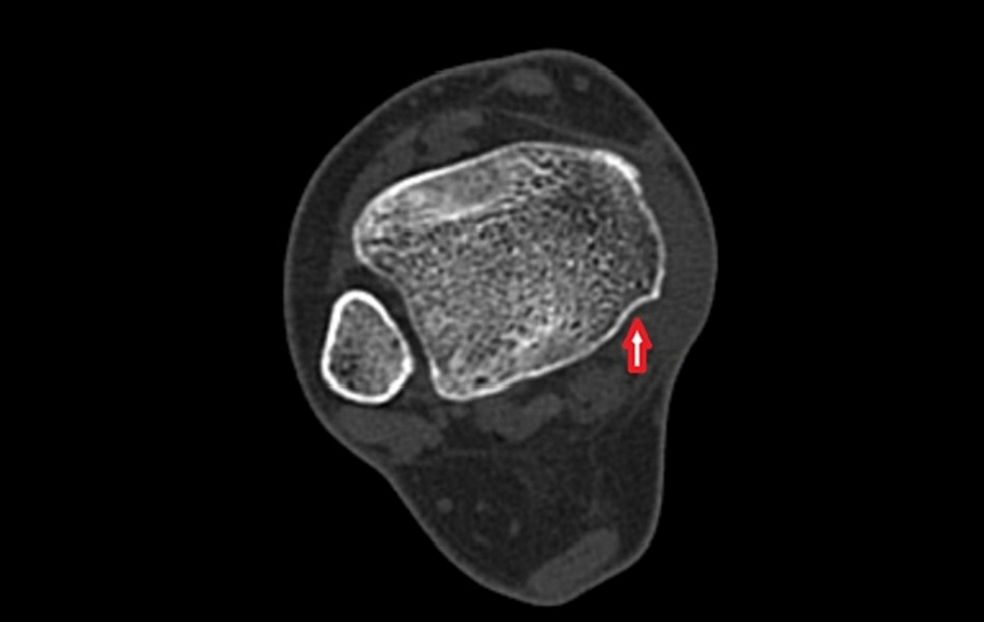
CT scan – axial image showcasing a slightly shallow retromalleolar sulcus (red arrow). CT: Computed Tomography

Four weeks later, clinical reassessment revealed swelling subsidence, but the patient referred to a persistent pain over the medial aspect of her ankle that impeded full-weight bearing and the return to everyday activities. A more detailed clinical examination unveiled a cord-like structure over the medial malleolus translating forwards and backward, with passive dorsiflexion and plantarflexion of the ankle. Computed Tomography (CT) did not showcase any bone participation, although a more flattened retromalleolar groove was depicted (Figure 1). Magnetic Resonance Imaging (MRI) of the right foot and ankle was carried out in order to exclude other pathologies of the surrounding ligamentous and bony structures.

**Figure 2:**
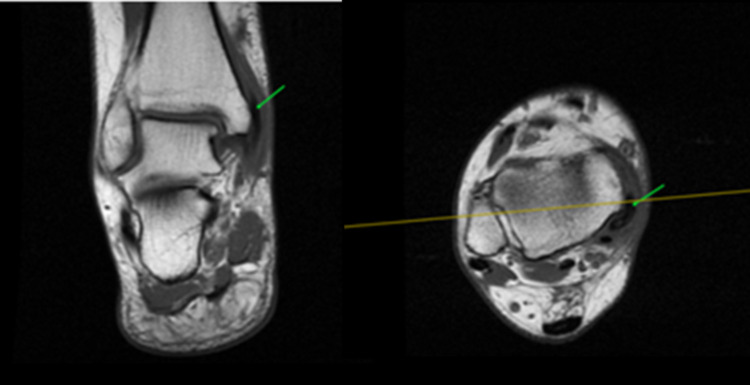
Ankle MRI-T1 sequence (coronal view-left, axial view-right) showcasing transposition of PTT (green arrow) over the medial malleolus. Yellow line indicating same anatomical level in axial compared to coronal plan. MRI: Magnetic Resonance Imaging PTT: Posterior Tibialis Tendon

MRI demonstrated the transposition of PTT over the medial malleolus, anteromedial to the retromalleolar sulcus (Figure 2), with excessive synovial effusion in the tendon sheath producing a focal high-intensity signal in STIR sequence. Concomitant bone bruise of the medial malleolus and adjacent soft tissue edema was noted. These radiological findings are highly indicative of PTT dislocation.

The patient was admitted to the Orthopedic clinic, and the surgery was carried out on the first-day post-admission. 

**Figure 3:**
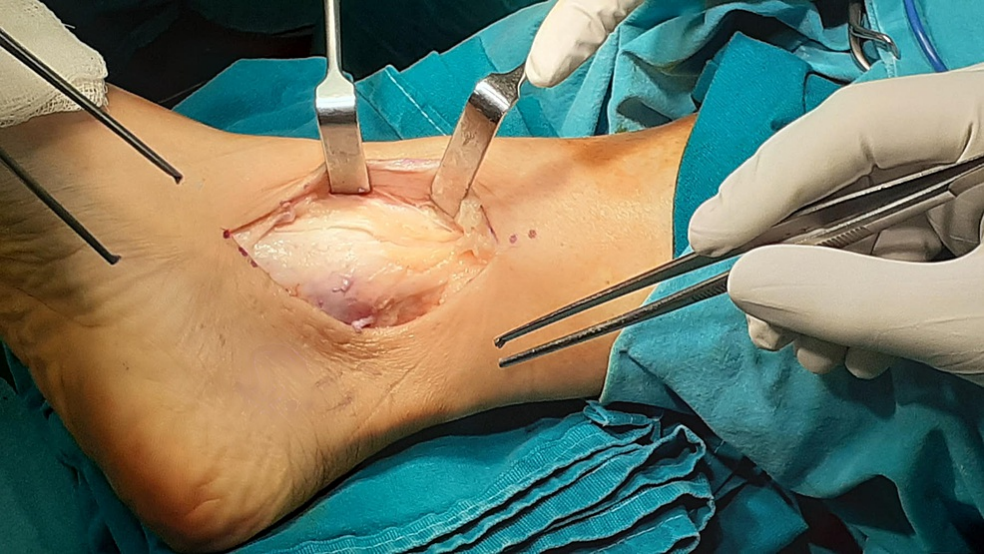
Skin incision: intact flexor retinaculum.

**Figure 4:**
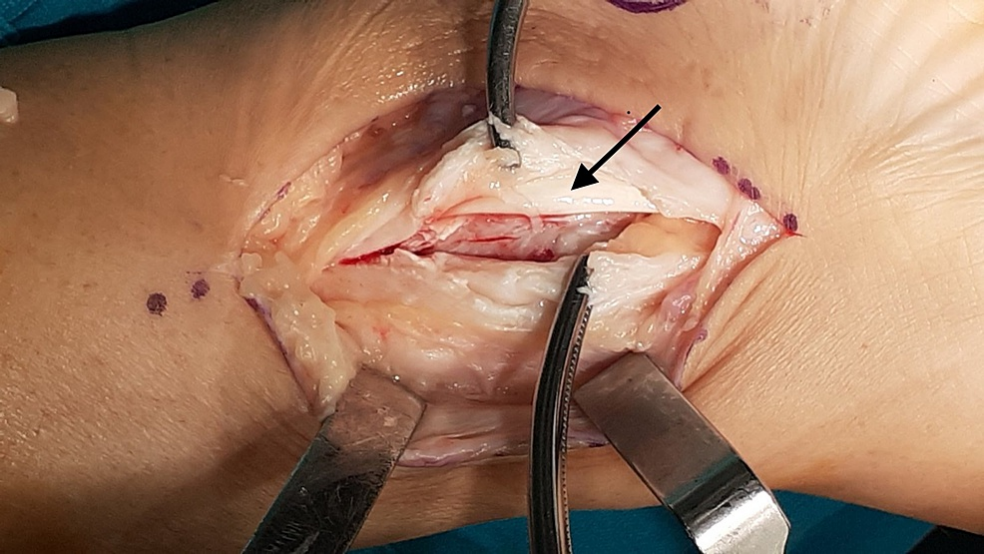
PTT (black arrow) settled in the pseudopouch over the medial malleolous. PTT: Posterior Tibialis Tendon

**Figure 5:**
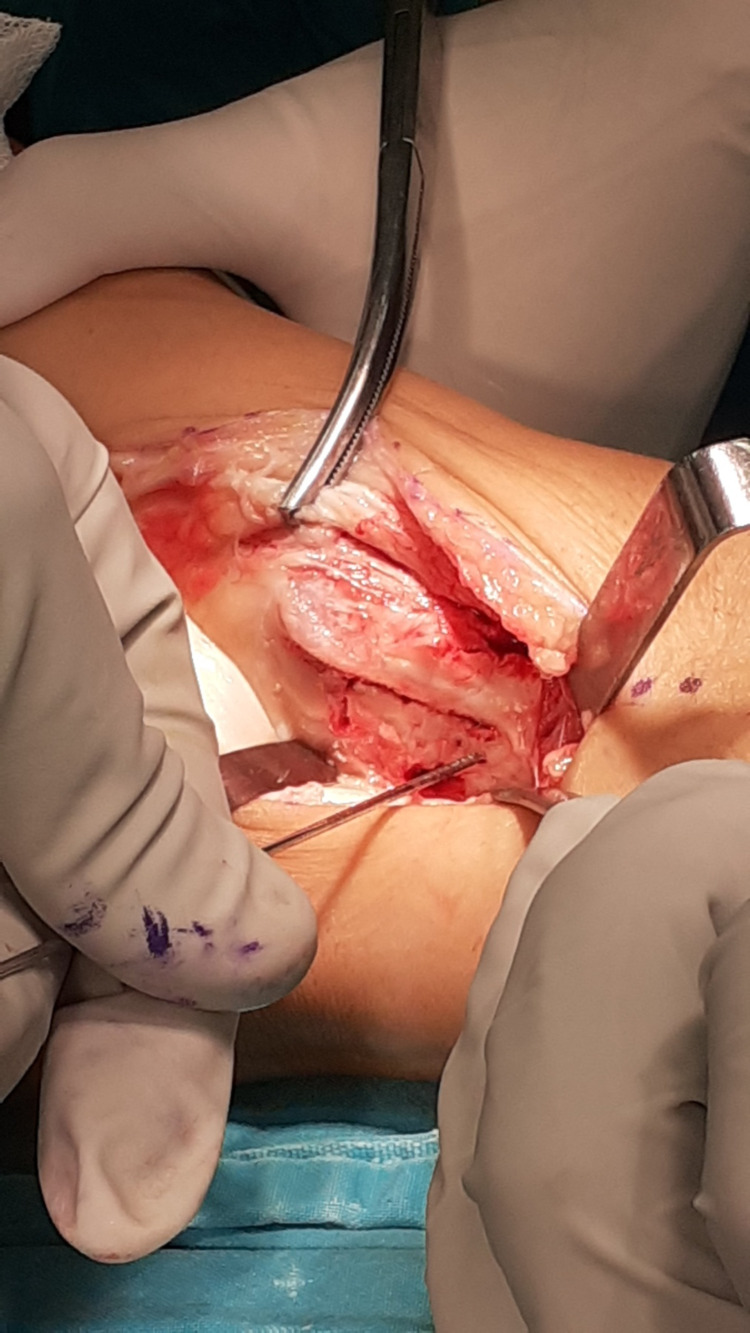
Formation of orthogonal cortical flap with drilling holes.

**Figure 6:**
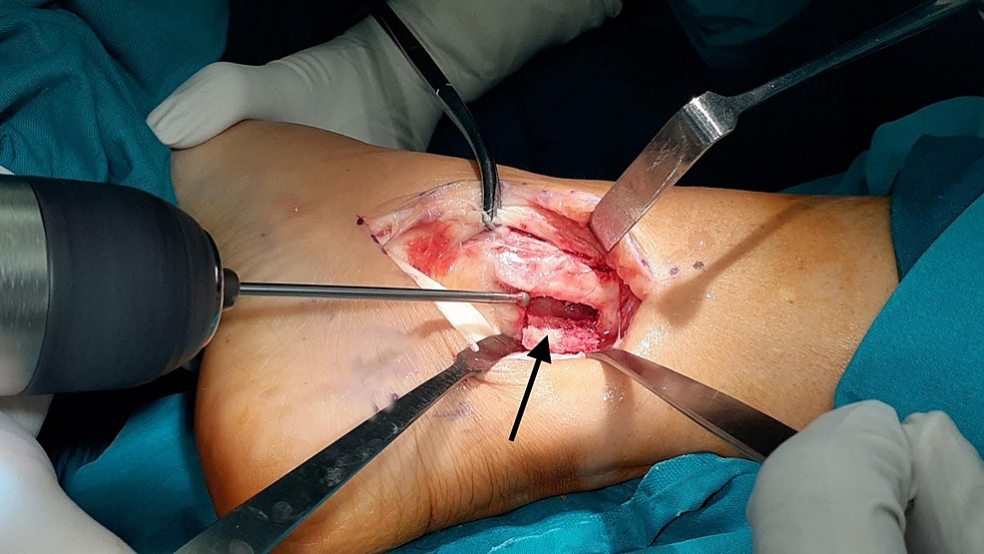
Orthogonal flap (black arrow): retromalleolar groove-deepening by recession of cancellous bone.

**Figure 7:**
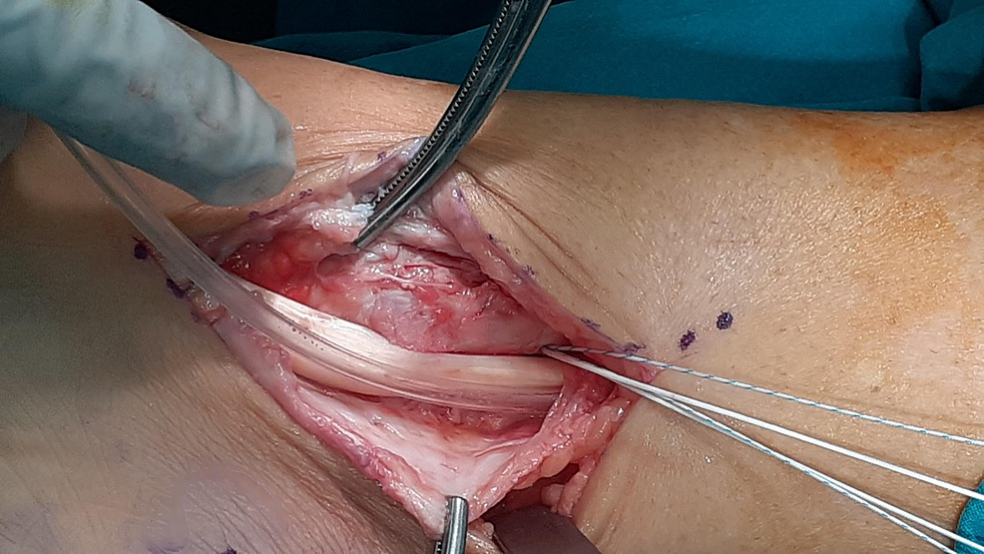
Relocation of PTT in anatomic position and suture anchor placement. PTT: Posterior Tibialis Tendon

**Figure 8:**
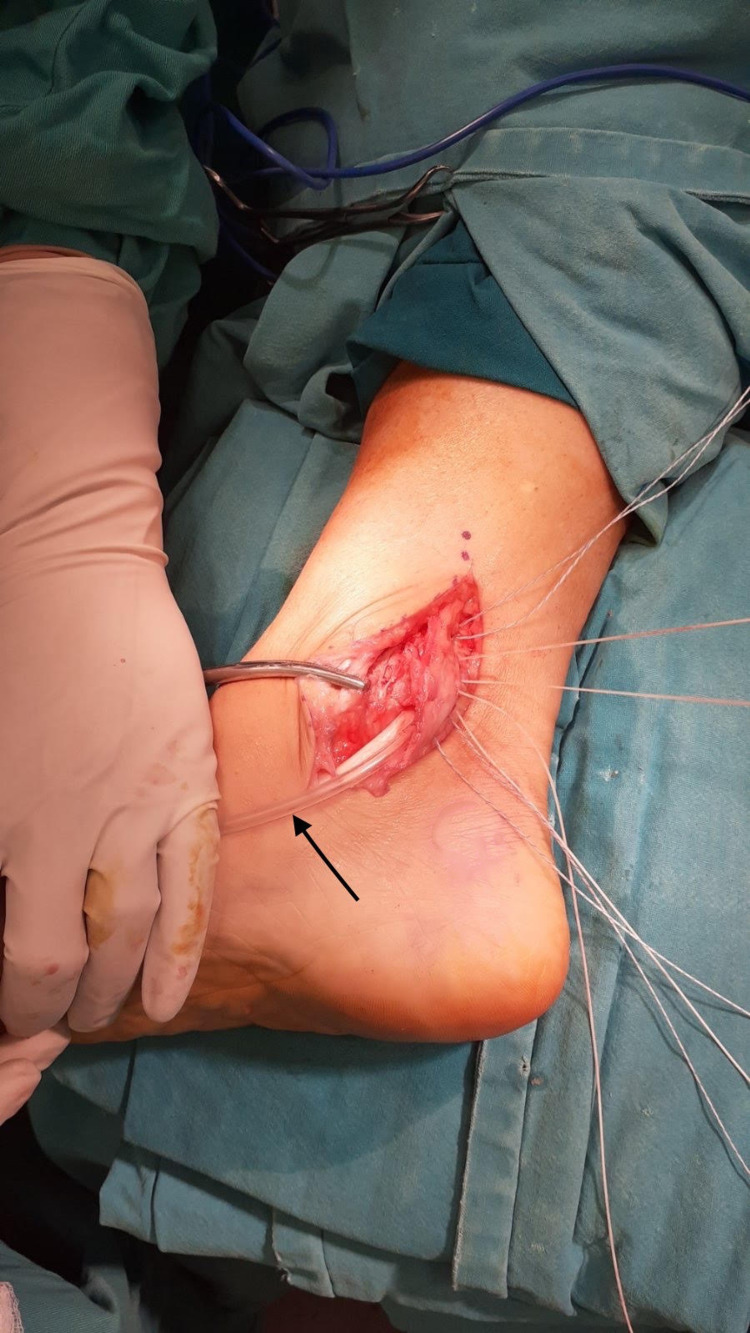
Restoration of flexor retinaculum: interposition of Nelaton catheter (black arrow) before suture tightening.

Under epidural anesthesia, with the patient placed supine on the operating table, a pneumatic tourniquet was applied around the upper thigh. The affected right foot was held in external rotation by an assistant. A 7.5 cm curvilinear incision was performed along the posterior border of the medial malleolus, followed by careful blunt dissection of subcutaneous tissue. The flexor retinaculum appeared intact (Figure 3). PTT was easily palpated and appeared to have migrated anteriorly over the medial malleolus. Flexor retinaculum was longitudinally incised 1 cm posterior to the anterior tibial retinaculum attachment in order to facilitate subsequent repair. The anterior aspect of the flexor retinaculum and periosteum was found avulsed from tibial bone, forming a false pouch in which the PTT was settled (Figure 4). These perioperative findings were consistent with a type II PTT dislocation. PTT was inspected, and no rupture of the tendon sheath was observed. Retromalleolar groove was judged hypoplastic intraoperatively; hence, a groove-deepening procedure was conducted before retinaculum repair in order to relocate the tendon in its anatomic position. Several drilling holes were made, creating an orthogonal cortical flap (Figure 5) that was recessed by using an osteotome. A generous amount of cancellous bone was removed with a rotary burr (Figure 6), and the cortical flap was repositioned and pulled into the deepened bony window by gentle hammering. PTT was manually repositioned in the deepened retromalleolar groove (Figure 7). Flexor retinaculum and periosteum repair were accomplished by the insertion of two intraosseous suture anchors in the anterior ridge of the retromalleolar sulcus, obliterating the pseudopouch. A 12-Fr Nelaton catheter was placed behind the PTT before suture tightening in order to ensure free gliding of the tendon after retinaculum fixation (Figure 8). Intraoperative passive dorsi- and plantar flexion, inversion, and eversion of the foot verified the maintenance and free gliding of PTT along the retromalleolar groove.

**Figure 9:**
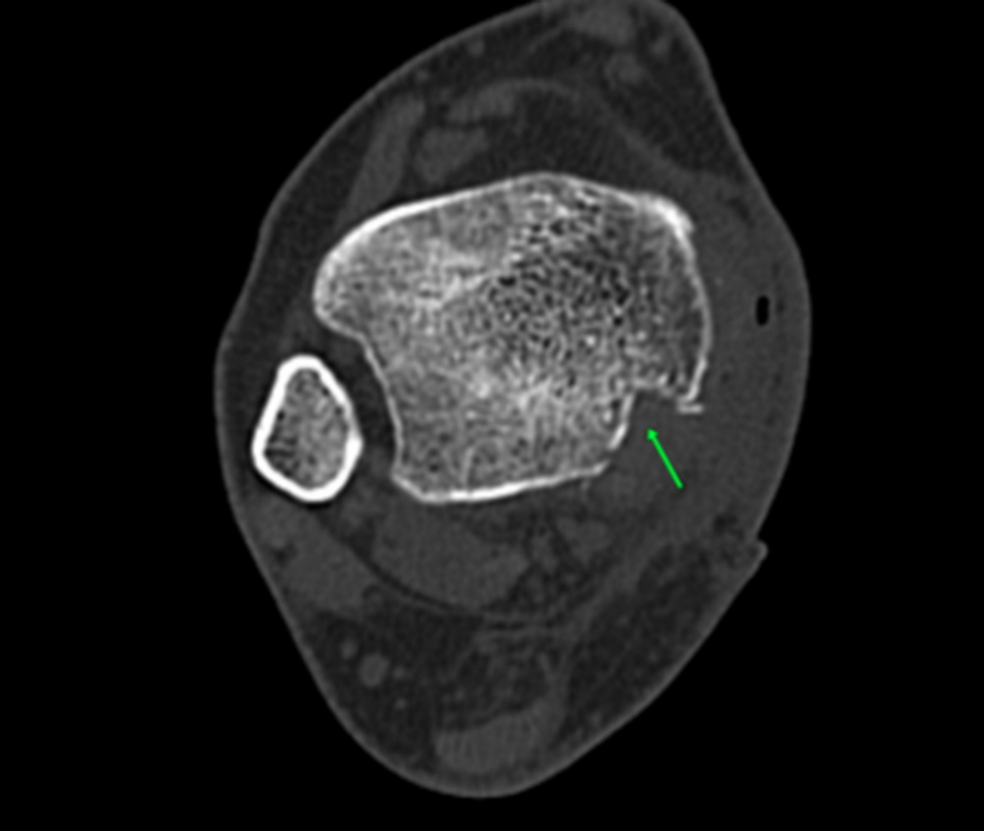
CT scan (axial view) of the right ankle depicting the deepened retromalleolar groove postoperatively (green arrow). CT: Computed Tomography

Early postoperative physical examination of the patient did not reveal any sensory/motor deficit or vascular compromise. A CT scan was conducted showcasing the newly formed retromalleolar groove (Figure 9). The patient was discharged home three days postoperatively with a lower leg back slab splint. Non-weight bearing mobilization with crutches was suggested for two weeks, along with straight leg raises. The following week, the back slab splint was switched to a controlled ankle motion (CAM) cast for three weeks, with the initiation of a gradual range of motion exercises and partial weight-bearing, as permitted. Full weight-bearing was allowed five weeks postoperatively coupled with ankle strengthening exercises and a proprioceptive training programme by a qualified physiotherapist. Follow-up duration was 12 months, with the patient's AOFAS (American Orthopaedic Foot and Ankle Society) Ankle-Hindfoot Score being 97/100 with a full range of ankle motion, no gait disturbances, and no limitation of everyday activities.

## Discussion

PTT dislocation was first described in Belgian literature by Martius in 1874 [4]. To our knowledge, statistical data about the incidence and prevalence of PTT dislocation are not available in the current literature. According to a recent systematic review by Lohrer et al. in 2008, 61 cases of PTT dislocation had been published until 2007 [5].

PTT is the most superficial structure of the tarsal tunnel. The flexor retinaculum, extending from the medial malleolus to the calcaneal tubercle, along with the concavity of the retromalleolar groove, is the main anatomic structure that holds the PTT and flexor tendons within the retromalleolar groove, preventing them from bowstringing. Soler et al. conducted an anatomic study on 25 dried cadaver shinbones and described that the retromalleolar groove can vary in width and depth, meaning that a shallower retromalleolar groove could make the luxation of PTT easier [6]. In our case, this was confirmed by the CT scan showcasing hypoplasia of the groove, thus facilitating PTT dislocation.

**Figure 10:**
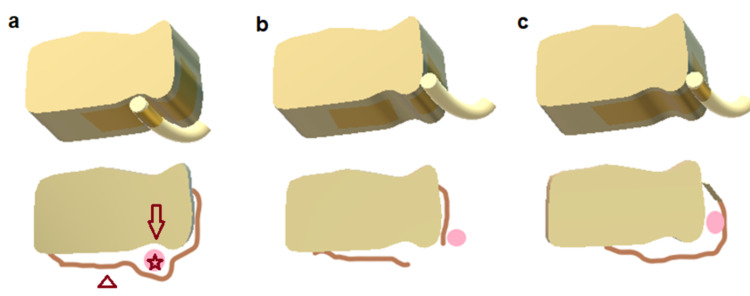
Schematic 2-dimension (2D) and 3-dimension (3D) representation of PTT dislocation classification (Redrawn after Sakakibara et al 2018) a) Anatomic position of PTT. b) Type I dislocation of PTT: ruptured flexor retinaculum c) Type II dislocation of PTT: avulsed flexor retinaculum/False pouch (Arrow: retromalleolar groove, Arrowhead: Flexor retinaculum, Asterisk: PTT). PTT: Posterior Tibialis Tendon

Typically, the preferred mechanism of injury is a twisting of the ankle, pronation-external rotation, or forced dorsiflexion, although non-traumatic cases of spontaneous PTT dislocation have been reported, as well [7,8]. Two types of PTT dislocation have been recognized. In type I, the flexor retinaculum is ruptured, thus allowing the PTT to freely move over the medial malleolus in the subcutaneous tissue. In type II, flexor retinaculum and periosteum are detached from the tibia, leading to the formation of a pseudopouch in which the PTT can settle (Figure 10) [9].

The diagnosis of PTT dislocation is mainly clinical; however, in an acute setting, the diagnosis can be delayed as the history and clinical presentation of the patient can mimic an acute ankle sprain [2]. Ouzounian et al. reported nine months as being the usual meantime from presentation to diagnosis [3]. During the physical examination, concomitant ankle edema can impede the palpation of the dislocated PTT. As ankle swelling subsides, a cord-like structure can be palpated over the medial malleolus. In doubtful cases, imaging modalities such as ultrasonography, MRI, and/or CT scan aid the establishment of an accurate diagnosis. Dynamic ultrasonography, other than being more cost-effective than a CT or MRI, can show the translation of the tendon in front and at the back of the malleolus [10]. CT scan can be useful in order to estimate the hypoplasia of the retromalleolar groove, while MRI can evaluate not only the PTT but also visualize the flexor retinaculum and possible tendon tears [6,11]. Plain x-rays in an acute setting aid primarily to exclude bone participation [9].

Treatment choices include conservative or surgical management. Up to date, literature acknowledges that the surgical approach is most effective to restore the dislocation [3,12-14]. According to the imaging and perioperative findings, several surgical procedures have been suggested; direct flexor retinaculum repair, reconstruction of the retinaculum combined with groove-deepening procedures, malleolar osteotomies, suture anchors, autogenous bone block grafting, and buttress plate fixation [1,10,15,16].

## Conclusions

PTT dislocation is a rare yet important finding in a patient presenting with trauma and swelling of the medial malleolus. Due to regional edema, PTT can be impalpable in an emergency setting and is usually considered as an ankle sprain leading to unsuccessful conservative treatment. The fact that the injury is not tackled in the acute phase can generate chronic conditions, recurrent dislocations, acquired flatfoot, or even tendon ruptures. Persistent pain on the medial malleolus should make the physician consider PTT dislocation as part of the differential diagnosis. A dynamic ultrasound, MRI, or CT will confirm the diagnosis. Treatment is mainly surgical, restoring the PTT to its anatomical position. In our case, PTT dislocation was attributed to a hypoplasia of the retromalleolar groove in combination with a sudden contraction of the tendon in a fully extended limb. Our treatment regimen aimed to deepen the retromalleolar groove and restore a competent flexor retinaculum, leading to an optimal functional outcome for the patient.
